# Listening to families with a person with neurodegenerative disease talk about their quality of life: integrating quantitative and qualitative approaches

**DOI:** 10.1186/s12955-022-01977-z

**Published:** 2022-05-07

**Authors:** Alba Aza, María Gómez-Vela, Marta Badia, M. Begoña Orgaz, Eva González-Ortega, Isabel Vicario-Molina, Estrella Montes-López

**Affiliations:** 1 Institute on Community Integration (INICO), Faculty of Psychology, University of Salamanca, Avda. De la Merced, 109-131, 37005 Salamanca, Castile and Leon Spain; 2 Institute on Community Integration (INICO), Faculty of Education, University of Salamanca, Salamanca, Spain; 3 Teacher Training College of Zamora, University of Salamanca, Zamora, Spain; 4 Department of Sociology and Communication, Faculty of Social Sciences, University of Salamanca, Salamanca, Spain

**Keywords:** Neurodegenerative diseases, Family Quality of Life, FQoLS-ND, Focus groups

## Abstract

**Background:**

The diagnosis of a neurodegenerative disease (ND) produces profound changes in the quality of life of the affected families. Despite the vital importance of these processes, the scientific literature has addressed this topic almost exclusively relating to the main caregiver or using limited approaches. Thus, the main objective of this research is to achieve a deeper understanding of the quality of family life of people with a neurodegenerative disease, following a mixed-method approach that combines quantitative and qualitative methodology.

**Methods:**

The quantitative instrument was the Spanish version of the *Family Quality of Life Survey-Neurodegenerative Disease* (FQOLS-ND)*,* which was completed by 300 participating families. The qualitative methodology was used in two focus groups with family caregivers, with a total of 21 participants.

**Results:**

On the one hand, confirmation of the dimensional structure of the scale in the focus groups was obtained and, on the other hand, the results of family quality of life in attainment and satisfaction were shown to be high for Family Relations and Careers and Planning for Careers and low for Support from Services and Leisure and Recreation.

**Conclusions:**

The results of this study, through the combination of quantitative and qualitative information, helps to identify key issues to optimize services that respond to the priority needs of families.

**Supplementary Information:**

The online version contains supplementary material available at 10.1186/s12955-022-01977-z.

## Background

Neurodegenerative diseases (ND) have a significant impact on the lives of people who experience them, producing a progressive deterioration in cognitive, physical, and social functioning. This leads to a growing loss of independence and an increase in care and support needs [1]. According to the World Health Organization [2], dementias are responsible for the greatest burden of ND and affect around 50 million people worldwide. In Spain, there are more than one million people affected by different NDs. Alzheimer's disease and other neurodegenerative conditions, such as Parkinson’s disease, multiple sclerosis, neuromuscular diseases, and amyotrophic lateral sclerosis, are the most prevalent, affecting 5.4% to 14.9% of people over 65 years [3]. In a recent epidemiological study [4] carried out in the rural cross-border area of Spain–Portugal, the prevalence of Alzheimer’s disease and other dementias was estimated at 4.5%, Parkinson’s disease at 2.7%, and multiple sclerosis at 0.03%.

The family is a major determinant of positive outcomes in people with ND. It provides emotional and practical support, social care, and assistance in the basic and instrumental activities of daily life [5]. Living with a person with ND is a huge challenge that can lead to negative consequences, among which are anxiety and depression, stress, burnout, and social isolation [6]. However, the caregiving role can also have positive effects such as greater closeness to the affected person, reciprocity, and spiritual growth [7]. When we refer to family, we adopt the broad sense of the word, meaning “people who are closely involved in the daily affairs of the home and support each other on a regular basis, whether they are related by blood, marriage, or a close personal relationship” [8, 9] and including the person with ND. In the last few years, the study of Family Quality of Life (FQoL) has increased, understood as a “dynamic sense of family well-being, collectively and subjectively defined and informed by its members, in which individual and family-level needs interact” (p. 246) [10]. It is a perspective that considers the family as an interconnected system, such that ND affects not only the person who suffers from it and their family members, but also the interaction that occurs between them [11, 12].

Like individual quality of life (QoL), FQoL is a multidimensional construct that includes both objective and subjective indicators, which has been primarily and effectively studied in the evaluation of the strengths and challenges of family caregivers of individuals with disabilities and chronic health conditions [13]. According to the FQoL theory, individual member concepts (i.e., demographics, characteristics, and beliefs) and family-unit concepts (i.e., dynamics and characteristics) are direct predictors of FQoL and they interact with individual and family-level supports, services, and practices [14].

Given the importance of the role of the family caregiver for people with ND [15], the family needs to maintain good levels of FQoL. Hence, the views of families are a starting point for the design of support plans [16] and the improvement of professional attention, services, policies, and state, regional, and local programs.

To monitor this process effectively, it is necessary to have psychometrically robust measures, and, as far as possible, specific for this population [1]. One of the most widely used scales is the Family Quality of Life Survey-2006 (FQOLS) Brown et al. 2006 [17], which considers nine life domains in terms of importance, opportunities, attainment, initiative, stability, and satisfaction. This scale was initially constructed for families caring for a family member with an intellectual disability but has been adapted and validated for other populations, such as ND [13, 18, 19].

However, FQoL also has subjective components, so it would be unwise to base its evaluation exclusively on quantitative data. Qualitative assessments are important because beliefs and attitudes are relevant for understanding caregivers' feelings and behavior [20]. In this regard, focus groups elicit perspectives of the targeted population on issues of concern through structured and facilitated discussion [21]. They are particularly useful in the analysis of specific factors in the life of the participants, such as FQoL [22, 23]. Some studies using focus groups [14] indicate that disability-related support, medical care, and physical and cognitive well-being were by far the most cited needs in this population.

The literature has focused on the QoL of family caregivers from the main caregiver’s point of view, paying less attention to the QoL of the whole family unit [24, 25]. It has also used either quantitative or qualitative models, seldom considering the positive contributions of the combination of both models through a mixed-methods approach [26–28].

Therefore, the objective is to study FQoL in families with one or more members with ND by using a mixed-methods approach, i.e., contrasting the application of a specific quantitative instrument for this population with the qualitative assessments made by the families using the focus group methodology. Specifically, we intend to (1) confirm whether the FQoL domains, as evaluated with the *Family Quality of Life Survey–Neurodegenerative Disease* (FQOLS-ND) [18], can be replicated qualitatively; and (2) obtain a deeper understanding of the attainment and satisfaction dimensions of the FQOLS-ND by comparing the quantitative and qualitative data.

## Method

### Participants

Participants in the quantitative assessment and those who participated in the focus groups were not the same.

Participants for the quantitative part were recruited by the Regional Health Management (RHM) of Castile and Leon (Spain), between October 2019 and July 2020. Family members of patients with ND were invited to participate if they met the following inclusion criteria: (1) contribute to the daily care of the person with ND but not necessarily as the primary caregiver; (2) 18 years of age or older; (3) live in the cross-border area of Spain-Portugal. Families whose member with an ND lived in residential accommodation were excluded. The RHM selected 890 families from the population (*N* = 987; Dementia: 58.7%, Parkinson: 37.7%, Multiple Sclerosis: 3.6%). All of them were provided with information about the research (e.g., importance of the study, objectives, tasks to be performed, ethical standards and data protection, potential benefits, etc.), collected in a model of informed consent that they had to sign. A total of 380 family members signed the informed consent to participate, but 74 of them were not accessible (*n* = 35), available (*n* = 2), or willing to collaborate (*n* = 37).

Participants for the qualitative part of the study were recruited between November 2019 and January 2020, with the support of the following associations: *Asociación de Familiares de Enfermos de Alzheimer (AFA Salamanca), Asociación de Familiares de Enfermos de Alzheimer de Béjar y Comarca (AFABECO), Asociación Parkinson de Salamanca* and *Asociación Salmantina de Esclerosis Múltiple (ASDEM)*. Family members of patients with ND were invited to participate if they met the same inclusion–exclusion criteria as the quantitative study. All those interested in participating received information (verbal and written) on the objectives of the study and the research technique to be used. None of the participants had comprehension or oral expression difficulties in Spanish. Finally, 21 people signed the informed consent form and participated in two focus groups (10 in the first and 11 in the second).

### Instruments

The study used both quantitative and qualitative methods. The rationale of this particular mixed-methods design is that quantitative instruments provide empirical information on the QoL of extensive groups of family caregivers of persons with ND and help to plan, implement, and measure the effectiveness of family-centered interventions [29], whereas qualitative methods allow researchers to talk directly with caregivers to learn about their experiences [30], perceptions, and the supports needed. Concurrently, combining quantitative and qualitative analyses allow us to corroborate the information gathered from both approaches and to achieve a deeper understanding of FQoL [28].

The quantitative instrument was the Spanish version of *Family Quality of Life Survey–Neurodegenerative Disease (FQOL-ND)* [18], translated, adapted, and validated from the *FQOLS–Dementia* [15]. Part A includes questions about the family and the person with ND, which range from general socio-demographic issues to more specific ones, such as the supports needed by the family member with ND or the degree of independence in daily life activities. Part B contains nine life domains (i.e., Family Health, Financial Well-being, Family Relationships, Support from Others, Support from Services, Influence of Values, Careers, Leisure and Recreation, and Community Interaction) to examine how the family perceives its FQoL. In the Spanish version, only the attainment and satisfaction dimensions for each of the domains have been considered and validated, because they are the two main outcome measures with the best psychometric results [13, 17, 18, 27]. These dimension items collect quantitative data on a 5-point Likert scale, with higher scores indicating higher levels of attainment or satisfaction in the specific domain. The final section consists of two close-ended questions about global impressions of FQoL. The internal consistency was strong for all domain subscales (from α = 0.80 to 0.91) and excellent for the total FQOL-ND scale (α = 0.85) and the Global FQOL-ND scale (α = 0.87) [18].

Using a qualitative perspective, two separate focus groups were conducted, and data were collected using guiding questions to identify core QoL dimensions for families of persons with ND, to obtain information on how ND affects FQoL, and to check and/or modify the nine domains of the FQOL-ND (Additional file 1: Appendix 1. Caregiver Focus Group Questions).

A protocol was designed to ensure the systematic development of two focus groups, considering the quality indicators of DiZazzo et al. [15]: greeting participants, a brief introduction to the goals of the research, informed consent, basic rules about the group’s functioning, and main topics (i.e., FQoL and the nine domains).

### Procedure

In the quantitative evaluation, the questionnaires were administered by telephone in about 30 min by trained and experienced interviewers to comply with the social distancing restrictions of the COVID-19 pandemic. Verbal and written informed consent was obtained after informing participants about the aim of the study and their right to drop out at any time.

In the qualitative study, the first focus group took place at the Association of Family Members of People with Alzheimer's in Béjar, a rural zone in Salamanca, and the second group at the Parkinson’s Association in Salamanca (Spain), both led by a researcher with training and experience. With the participants’ agreement, a voice recorder was used, and a co-researcher was present, who took notes to ensure data collection consistency across groups and to minimize data loss. The focus groups were conducted using the guiding questions (Additional file 1: Appendix 1) in about 2.5 h.

The study was approved by the Bioethics Committee of the University of Salamanca (Protocol No. 2019/238). All procedures comply with the principles of the 1964 Declaration of Helsinki and its amendments. Verbal and written informed consent was obtained from all participants prior to data collection.

### Data analysis

Quantitative data were analyzed using SPSS 26. The statistical significance was set at *p* ≤ 0.05. Descriptive data were displayed as the mean, Standard Deviation (SD), and range interquartile or absolute and relative frequencies (i.e., percentages). Next, repeated-measures ANOVAs were used to compare the nine domains, the two dimensions, and the interaction between domains and dimensions.

In the qualitative analysis, the transcribed texts of the focus groups were analyzed by means of Atlas.ti 7. A deductive method was used, that is, a codebook was created based on the literature about FQoL. Specifically, the construction of this codebook was based both on the use of publications about ND [13, 38] (Ducharme et al. 2014) and publications about FQoL in population with other disabilities [10, 27, 28], given the limited number of specific literature in the field of ND. This previous step allowed us to guide the process of encoding the information, keeping in mind the comprehensiveness of the data and the objectives of the study (i.e., content analysis). This codebook was developed by two of the team's researchers with the most experience in the field of FQoL and in qualitative methodology. Finally, it was composed of 17 categories and more than 100 structured codes following the nine domains of the FQoL-ND scale.

The analysis began with the identification of text segments suitable for coding. These fragments were relatively large to allow embedding them in their logical context, and therefore parts of them were selected. A tree structure (i.e., domains, categories, and codes) was gradually refined and attuned to clarify the definitions of categories and subcategories. To do this, in a first phase, one member of the research team, who had participated in its creation, verified the belonging and usefulness of the initial codebook through its application in a sample of focus groups. This made it possible to complete the codebook with quotes taken from these groups. It also enables us to make code adjustments and even incorporate some new codes that emerge inductively. In a second phase, the coding of the rest of the focus groups was carried out with the modified codebook. To do this, an external person with knowledge in the field was hired and received specific training in the coding process, which was supervised and reviewed by the member of the research team.

## Results

### Sample characteristics

The final sample for the quantitative part of the study consisted of 300 participants (mean age = 62.4, *SD* = 13.34). The majority were females, married/with a partner, not working, with low income –up to 1000 EUR per month– and had primary or secondary school qualifications. Most of them were the spouse/partner or son/daughter of the person with ND, they had the role of primary caregiver and lived in the same household. Most of them lived in rural areas of up to 500 or 500–10,000 people (Table 1).

**Table 1:** Family caregiver characteristics (*N* = 300)

Variable	*N*	%
Age (*M* = 62.48, *SD* = 13.34, Range = 25–88)		
Up to 65 years	178	59.3
More than 65 years	122	40.7
Gender		
Male	90	30.0
Female	210	70.0
Educational level		
No school certificate	21	7.0
Elementary school	150	50.0
High school	68	22.8
University	59	19.8
Employment status		
Working (employees + self-employed)	106	35.3
Not working (retired + unemployed + others)	194	64.7
Income (EUR per month)		
Up to 500	95	31.9
500–1000	102	34.2
1000–1500	69	23.1
More than 1500	32	10.7
Marital status		
Married or with partner	239	79.7
Others (divorced/separated, widowed, single)	61	20.3
Place of residence—number of inhabitants		
More than 10,000	61	20.3
500–10,000	107	35.7
Up to 500	132	44.0
Relationship with the person with dementia		
Spouse or partner	117	40.9
Son/Daughter	148	51.7
Others	21	7.3
Primary caregiver		
Yes	280	93.3
No	20	6.7
Living condition		
Living with the patient	225	75.0
Not living with the patient	75	25.0

As for the characteristics of the care recipients, their mean age was 79.3 years (*SD* = 11.7), and most were females. The majority had a diagnosis of dementia with some level of dependence, generally high or moderate (Table 2).

**Table 2:** Care-recipient characteristics (*N* = 300)

Variable	*n*	%
Age (*M* = 79.3, SD = 11.7, Range = 20–98)		
Gender	120	40.0
Male	180	60.0
Female		
Diagnosis		
Dementia	163	54.3
Parkinson’s Disease	80	26.7
Multiple Sclerosis	20	6.7
Others (unknown by family; several NDs)	37	12.3
Dependence		
Yes	202	67.3
No	98	32.7
Degree of dependence		
Degree 1	46	23.1
Degree 2	61	30.7
Degree 3	92	46.2
Supports needed		
None	27	9.0
Very few	32	10.7
Some	75	25.0
Quite a lot	56	18.7
A lot	110	36.7
Communication skills		
Poor communication	76	25.3
Only basic needs	26	8.7
Needs, desires, ideas	42	14.0
Coherent on some topic	66	22.0
Coherent on many topics	90	30.0

For the qualitative part of the study, two focus groups were formed, one composed of 10 and the other of 11 participants. The caregiver's age ranged between 40 and 79 years. The majority were females, spouse/partner of the care-recipient, and they had the role of primary caregiver (Table 3).

**Table 3:** Descriptive Statistics for Caregivers and Care Recipients in focus groups

*Caregivers’ sociodemographic data* (*N* = *21*)	*N* = 10 Dementia	*N* = 11 Parkinson
*Caregiver’s age in years* Age (Range = 40–79)		
Caregiver’s gender		
Female	7	10
Male	3	1
Caregiver’s role		
Primary caregiver	7	9
Shared primary caregiver	3	2
Caregiver’s relationship		
Spouse	6	9
Sibling	1	1
Son/daughter	3	0
Daughter-in-law	0	1
*Care recipient’s sociodemographic data (N = 21)*		
Care recipient’s age in years (≥ 70 years)		
Care recipient’s gender		
Female	6	9
Male	4	2

### Confirmation of FQOL-ND structure

In previous publications, it has been shown that the structure of the Spanish version of the FQOL-ND [18] reproduces the original scale [15].

In this study, all the domains included in the FQOL-ND scale were mentioned in both focus groups, thus confirming their structure. Additional file 2: Appendix 2 (Categories and codes analyzed in each dimension and times mentioned in each focus group) shows in detail the categories and codes analyzed in each dimension and how many times they were mentioned in each focus group. Questions could not be framed within any of the domains of the scale, for example, aspects related to gender (“*Society itself imposes excessive psychological dependence, especially for women. Therefore, you feel guilty if you do not do certain things, so you shame yourself into doing them”*) and love and affection as an explanation of care (*“We have ‘endured’ it, but I do not want to say it in quotes, because I do it with love”*).

The analyses of the information obtained in the focus groups revealed that Support from Services (32.7%) and Family Health (31.5%) were the most frequently mentioned topics, and the dimensions with the lowest number of mentions were Support from Others (1.4%) and Careers and Planning for Careers (0.8%).

### Domains and dimension’s analysis

The mean scores, medians, and standard deviations for attainment and satisfaction in the nine domains are shown in Table 4, as well as the global and general scores. Table 5 includes the response percentages for each of the Likert-type options.

**Table 4:** Descriptive results of the FQOLS-ND

Domains	Dimensions					
	Attainment			Satisfaction		
	Mean	Median	SD	Mean	Median	SD
Family health	3.64	4.00	0.69	3.66	4.00	0.84
Financial well-being	3.53	4.00	0.64	3.58	4.00	0.69
Family relationships	4.41	5.00	0.72	4.35	5.00	0.80
Support from others	3.09	3.00	1.26	3.72	4.00	0.94
Support from services	2.83	3.00	1.02	3.11	3.00	1.11
Influence of values	3.49	3.00	0.86	3.64	4.00	0.82
Careers and planning for careers	3.96	4.00	0.71	3.98	4.00	0.75
Leisure and recreation	3.25	3.00	0.98	3.43	4.00	0.95
Community interaction	3.74	4.00	0.80	3.83	4.00	0.71
Global family quality of life	3.52	3.50	0.49	3.71	3.80	0.49
General family quality of life	3.65	4.00	0.70	3.69	4.00	0.47

**Table 5:** Distribution of frequency of scores in satisfaction and attainment for the nine domains of the FQOL-ND

Domains	Attainment					Satisfaction				
	None (%)	Very little (%)	Some (%)	Quite a lot (%)	A lot (%)	Very dissatisfied (%)	Dissatisfied (%)	Neither satisfied nor dissatisfied (%)	Satisfied (%)	Very satisfied (%)
Family health	0	6	29.7	58.3	6	1	9.7	22.3	56	11
Financial well-being	0.7	4.3	37.7	56	1.3	1	6.3	28	62.7	2
Family relationships	0.3	0.7	9.3	36.7	53	0.7	2.7	8.7	37.3	50.7
Support from others	14	13.6	30.3	33.7	8.3	1.3	7.4	30.3	42.3	18.7
Support from services	13.7	19	40.7	24.3	2.3	12.7	13.7	28.7	40	5
Influence of values	2.7	3.7	48.3	32.3	13	1.7	2.3	41	40	15
Careers and planning for careers	0.3	3.3	15.7	61.7	19	1	4	11	64	20
Leisure and recreation	6.7	14	31.7	43.3	4.3	5.3	10.7	25.3	52.7	6
Community interaction	1.3	6.7	20	60.3	11.7	0.7	4	19	64.7	11.7
Global family quality of life	0.7	5.7	26.7	62	5	1.3	7	18.7	67.3	5.7

ANOVA revealed significant differences between the domains, *F*_(8, 292)_ = 96.77, *p* < 0.001, η_P_^2^ = 0.25. The significantly higher domains were Family Relationships and Careers and Planning for Careers, whereas the significantly lower ones were Support from Services and Leisure and Recreation (*p* < 0.001). Results indicated a significant difference between the dimensions, *F*_(1, 299)_ = 118.96, *p* < 0.001, η_P_^2^ = 0.29. The mean level of satisfaction was significantly higher than that of attainment. The interaction Domains X Dimensions was significant, *F*_(8, 292)_ = 32.69, *p* < 0.001, η_P_^2^ = 0.10, and the post hoc tests revealed that the mean level of satisfaction was higher than that of attainment in eight of the nine domains, although these differences were only significant (*p* ≤ 0.001) in Support from Others, Support from Services, Influence of Values, Leisure and Recreation, and Community Interaction.

The relatively high mean scores across dimensions and domains suggest that the participating families generally considered their FQoL as moderate to high. The SD showed considerable variation in individual scores. On another hand, in the focus groups, the QoL of the primary caregiver was generally adversely affected after the onset of ND, as also occurred in the rest of the family. However, resilient skills focused on disease acceptance also emerged:

For me, quality of life is normalization. We cannot return to the normality that we had before because they [the member with ND] present loss of balance, dizziness, they fall asleep, their vocalization fails a lot but I think that, concerning quality of life, we must focus on acceptance. The better we accept our situation, the better we can deal with it, so we do not get exhausted or wear them [the member with ND] out.

And we take it with humor. Let's see, I think it's very dramatic, but we have to laugh. I find laughing so basic.

Next, and following the structure of the mixed methods, an individual analysis of those areas in which more extreme scores were reported is carried out (i.e., the domains of Family relationships and Careers and planning for careers which reported the highest mean scores, and the domains of Support from services and Leisure and Recreation which reported the lowest). This selection of domains offers the objective of including an exhaustive analysis, adjusted to the publication space, and that allows to know in depth the reasons that lead to consider these areas as strengths and weaknesses.

#### Family relationships

Concerning family relationships, there were high levels of achievement (*M* = 4.41, *SD* = 0.72) and satisfaction (*M* = 4.35, *SD* = 0.80), in most cases (50.7%) reported as *“very satisfactory” or “satisfactory”* (37.3%). At the qualitative level, participants mentioned the unavailability of other family members (e.g., siblings of the caregiver or the person with ND) to help with the ongoing tasks. This was mainly due to work or geographical remoteness and hence, the responsibility fell on the main caregiver*.* However, the existence of timely supports or supports that do not require the physical presence of other family members was also mentioned, which contributed to FQoL in family relationships:

In our case, my sister lives farther away so she cannot come every day to take care of her [the family member with ND]. But we go together to the meetings of the care association and support my mother.

There was a duality in some cases where the presence of the ND constituted a family crisis (*"Well, in your case, she [the patient] is your wife, but in my case, it is my brother. I have other brothers. One brother understands it [the situation], but the other does not and says: ‘Well, just to leave it.’ How can just leave the situation? How am I going to leave it if he [the patient] is my brother?”*) but in other cases, ND contributed to the family’s union (“*The same as I tell you that it has united the family a lot, I also tell you that our friends have disappeared”).*

#### Careers and planning for careers

Less than 20% of families showed some level of dissatisfaction with their job. However, there was a high percentage of family members who were retired (64.7% of our sample was not working, was retired, or unemployed). In fact, in the case of actively working people, ND had a significantly negative effect both on the person with ND and on the other family members, either because they had to give up their jobs or their work obligations were incompatible with the care tasks at the long term:

Of course, it [caring for my family member] is already starting to affect my job.

#### Support from services

The satisfaction, and especially attainment, of Support from Services was very low (*M* = 3.11, *SD* = 1.11; *M* = 2.83, *SD* = 1.02, respectively). Concerning the health care of people with ND and their families, there were some complaints about the treatment by the primary care teams and specialized health care services. Moreover, access to a specialized professional or receiving their continuous attention was considered especially problematic. There was also a lack of information about the diagnosis and progression of ND and the needs of the patient:

Another thing I am concerned about is that family caregivers, in this case, husbands or wives, do not receive any help, either psychological or motor. How should you move your husband? You don’t know anything about posture, you are just messing about. How should you treat him?

However, cases of high satisfaction with specialized care professionals were also reported, highlighting the importance of feeling heard and cared for as a priority.

Among the social care services, the psychological and technical support, both for the family and the person with ND, and the presence of resources such as day centers/associations were highlighted as a need. On another hand, residential centers were considered as an option when the disease was more advanced or when the family could not take care of the person with ND. In addition, significant differences were found in the services associated with the person’s diagnosis:

I consider Parkinson's disease as the big loser. The big loser or the big forgotten. When you talk about Parkinson's, well, it means that the person stays at home and gets the tremors... I mean, that is the least... But when you mention Alzheimer's, there are residences for Alzheimer's, there are aids…

Regarding the barriers encountered in access to services, family members highlighted the scarcity of resources (in general and particularly in the rural environment), the lack of public subsidies, the high cost of private resources, the short duration or intensity of care, with patient overloads resulting in lower-quality care or long waiting times to access the resources they needed:

I think the medication does not control it [the ND] very well. I also detect patients who should have a much more timely follow-up, who do not have it. In fact, he [the person with ND] had problems with medication... I asked for an earlier appointment but nobody listened to me.

Therefore, they proposed the creation of more resources in the rural environment and more associations of caregivers, better access to specialized professionals, more information about the procedures to access resources and grants, and a greater number and intensity of activities to be carried out with the person with ND.

According to participants, the availability of resources would improve FQoL and would not only be a way to achieve respite in the task of care but also an opportunity for the family to establish new social ties:

…where we can meet, just like we are doing here. It is going to take a while and it looks like your problem has already improved a little bit. At least you see you are not alone.

#### Leisure and recreation

The domain of Leisure and Recreation was scored low both in attainment (*M* = 3.25, *SD* = 0.98) and satisfaction (*M* = 3.43, *SD* = 0.95). In fact, the relatives mentioned that, ever since the family member had the ND, their leisure had been reduced, but they maintained some activities both at the family level and with people outside the family:

What we are seeing is dependence, not the sick person’s dependence but the families’. The families become so totally absorbed, and their dedication is so exclusive that they give up what they used to do, their friendships, their outings, their cultural activities, their leisure time.

My eldest son says Mom, I will give you 4 h on Tuesday for you to go to the hairdresser or out with a friend…

Although the care of the person with ND was perceived as highly demanding in terms of time, the good disposition of the other members of the family provided some respite for the main caregiver who devoted more daily time to the person with ND.

## Discussion

This mixed-methods study attempted to explore how various domains and dimensions of FQOL are affected when the family has an individual with ND. The findings both from quantitative and qualitative data converge in supporting the approach of nine dimensions, showing that the lives of family members are affected by their caregiving role.

The participating families considered their QoL to be moderate to high. However, considerable inter-family variability was found, highlighting the importance of the FQoL approach, which is characterized by interventions that meet the specific needs of each family [14, 31, 32]. This perspective considers the family as possessing strengths and focuses the intervention on empowering and promoting competence and other positive aspects of family functioning [33].

Despite the impact of caring for a person with ND in important aspects of family life such as health, leisure time, work and plans, and expectations, the participants rated their FQoL as quite satisfactory, a finding also observed in studies with caregivers of individuals with developmental disabilities and dementia [13, 34]. Also, in terms of attainment and satisfaction*,* caring families reported high rates in Family and Careers but low rates in Leisure and Recreation and Support from Services. These results are consistent with other studies suggesting the positive effect of high-quality family relationships and the limited effect of caring for the person with ND on Careers and Planning for careers [13, 34]. There were also difficulties in accessing adequate professional services and enjoying leisure activities [15, 35, 36].

FQoL domains were explored in greater depth using focus groups. Regarding health aspects, the families reported that the behavioral problems, memory disorders, and limitations in communication presented by the person with ND affected family well-being. Consistently, several studies have shown that behavioral and cognitive alterations produce significant levels of discomfort in the caregivers [14, 37–39]. Likewise, the main caregivers complained about their psychological well-being, associated with overload, inability to disconnect, feelings of loneliness and discomfort linked to the suffering of their family member, factors widely confirmed by the previous literature [38, 40–42]. At the same time, they underlined that the care required by the person with ND also affected the psychological well-being of other family members [43].

Caring for a person with ND implies a detriment to family finances. The families stated that they did not have sufficient financial support to adequately cover the costs of caring for the person with ND (e.g., conditioning the home to the needs of the person with ND). In Spain, the family is the great provider of support resources, and this implies no cost to the public health and social services system but a very high cost for the families [3]. In addition, the family careers of people living in rural areas are at a greater disadvantage, generally receiving lower incomes [44].

An important aspect that contributes to FQoL is the support families receive from others. The families reported that they did not receive emotional and practical support from their extended family, friends, or neighbors. This situation produces feelings of loneliness and social isolation. These results confirm previous studies that highlight the negative consequences of the lack of informal supports for the family [13, 34, 38, 45, 46].

Regarding how the person with ND has conditioned family relationships, the participants stated that they were satisfied, suggesting that families can generally make the emotional adjustment to understand and accept the disease and its care [11]. In our study, the responsibility for the care of the person with ND lay mostly with the main caregiver due to the work duties or the distant geographical location of the other members of the family. However, this does not prevent their relationships from being supportive, respectful, and of mutual trust. In fact, Losada-Baltar et al. (2017) [47] concluded that family cohesion, the family's ability to communicate effectively, express emotions, and adapt to changes are essential for good family functioning.

In general, the families reported that they were dissatisfied with the professional support received, showing that the services provided are insufficient and poorly organized. In Spain, attention to dependency in the public system of social services, especially the services that support inclusion in the community and the respite of family members, is very limited [48]. This shows that the care for the person with ND falls on the family, especially, the women. Similarly, the participants expressed their difficulties in accessing social and health services appropriate to the needs of the patient and the family. For example, they highlighted the lack of resources in rural areas, the lack of information about the disease, the long waiting times to access economic benefits linked to dependency, or the low frequency and coverage of home-help. These barriers have also been identified in several studies conducted with caring families living in rural areas [36, 49, 50]. However, the families in this study highlighted the relevant role of the third sector in supporting services, both for the person with ND and the family. Thus, they acknowledged the role of associations of relatives in the provision of services that the public sector cannot cover [51, 52].

This full dedication to caring also implies fewer opportunities for leisure and recreation [13, 35, 38, 45]. However, some comments indicated that family members offer practical and emotional support so that the main caregivers can have their own recreational spaces. Several studies have shown the positive effect of leisure activities on carers’ emotional well-being, particularly social activities, and participation in self-help groups [53–55].

In the focus groups, comments were made about the lack of information about ND in people of the community, the difficulties concerning how a person with ND should be treated, as well as the presence of compassionate or negative attitudes. This is in line with the work of Losada et al. [47], who pointed out that part of society’s lack of information about ND can cause behaviors of rejection, avoidance, or ignorance about how to act in certain situations. However, our participants acknowledged the importance of the support provided by the associative movement to face these adversities. Participation in a self-help group improves FQOL outcomes, buffers the impact of the burden of care, offers social support, gives opportunities to share experiences, and stimulates social relationships [46, 53, 54, 56].

This study has numerous strong points associated with the complementary use of quantitative and qualitative information in the study of the FQoL of patients with ND. However, it is not without limitations. Firstly, one of the focus groups included families that lived in Salamanca rather than in rural areas and, given the statistically significant differences found between the two groups in the support of the services, more focus groups are needed to expand the information and facilitate the comparison. Secondly, the families that completed the FQOL-ND scale were not the same as those that participated in the focus groups, although the sociodemographic characteristics were the same. Thirdly, on many occasions it was not possible to have family members who were not the primary caregivers. In this sense, it was a substantial advance to include an analysis of the changes in all the relatives, as well as in their roles, interactions, etc. but it would have been even better if that information had been reported too by family members who were not the primary caregiver. It would even have been interesting to collect information from more than one member of the same family. In future research, it would be interesting to include more focus groups to study the influence of certain factors on FQoL levels.

## Conclusions

In conclusion, this mixed-methods study provides important information about how a person with ND affects families, what these families need, and how to optimize the supports provided. Thus, we found areas, such as family relationships, that were positively affected compared to others rated more negatively, such as the professional services received or leisure activities. The quantitative study provides reference values of the different quality of life domains, and the focus groups have contributed many details that numbers cannot provide.

## Supplementary Information

**Additional file 1: Appendix 1**. Caregiver Focus Group Questions.

**Additional file 2: Appendix 2**. Categories and codes analyzed in each dimension and times mentioned in each focus group.

## Data Availability

Data sharing is available upon reasonable request. Kindly contact the corresponding autor.

## Abbreviations

ND: Neurodegenerative diseases
FQoL: Family Quality of Life
QoL: Quality of Life
RHM: Regional Management of Health
