# Supplementary material for: Listening to families with a person with neurodegenerative disease talk about their quality of life: integrating quantitative and qualitative approaches
Source: Health Qual Life Outcomes. 2022 May 7;20:76. doi: 10.1186/s12955-022-01977-z (PMC9077340; doi:10.1186/s12955-022-01977-z)
Supplement: Supplementary file 1 — Additional file 1: Appendix 1. Caregiver Focus Group Questions. [file 12955_2022_1977_MOESM1_ESM.docx]

**Appendix 1**: Caregiver Focus Group Questions

| If I refer to FQoL: What do you think? What comes to your mind? To what do you think I'm referring? |
| --- |
| How would you define your FQoL? |
| FQoL may be affected by numerous events or situations, such as the presence of a health problem in a family member: Is your family member's health problem affecting the FQoL (as a whole)? If so:   - What aspect/s? (dimensions) - How? - Why? |
| Can you delve into how and/or to what extent you are being affected, in general, ...?   - Your health or physical status - Your mood or emotional status - Your family relationships (living together, communication...) - Your social relationships (with neighbors, friends, colleagues, etc.) - Your sex and/or partner life - Your economic situation - Your studies and/or working status - Your time and leisure activities - Etc. |
| Are any other family members also affected? If so:   - Who? - How? - Why? |
| What kind of support do you provide among family members in daily basic activities (e.g., medication, personal care, medical appointments)? |
| Do you receive financial support? If so, what kind? Or which one? |
| Do you receive professional support? If so, what kind? Or which one? |
| Do you receive support from other people (friends, neighbors, relatives...)? If so, what kind? Or which? |
| In what areas/aspects do you need the most support? (e.g., physical, emotional health, economic situation, etc.) |
| What would you need to improve the FQoL? |
