# Supplementary material for: Listening to families with a person with neurodegenerative disease talk about their quality of life: integrating quantitative and qualitative approaches
Source: Health Qual Life Outcomes. 2022 May 7;20:76. doi: 10.1186/s12955-022-01977-z (PMC9077340; doi:10.1186/s12955-022-01977-z)
Supplement: Supplementary file 2 — Additional file 2: Appendix 2. Categories and codes analyzed in each dimension and times mentioned in each focus group. [file 12955_2022_1977_MOESM2_ESM.docx]

**Appendix 2:** Categories and codes analyzed in each dimension and times mentioned in each focus group

| **Domains** | **Examples** | **Categories** | **Codes** | **Group 1: AFABECCO** | **Group 2:**  **Parkinson Salamanca** | **Total** |
| --- | --- | --- | --- | --- | --- | --- |
| Family health | *For example, I have a problem and I'm turning it over in my mind.* *I go to bed and I want to forget about it. But no, I'm continually thinking I have to do this, I have to do this. So I'm very stressed in that aspect because everything goes through my hands... if I have to keep an eye on it. And at night, I also have sleep apnea*.  *I know I should go for a walk with him. We didn't go out ... you see how I arrive at the center and that is like going from here to the park entrance, ... because he tells me we're going alone, and I say no, I have pain. I always say that, why? I can't, I can't walk, that the thing would best suit me, if anything.* | Health of the person with ND | Behavioral disturbances | 20  8.5% | 9  5.8% | 29  7.5% |
|  |  |  | Memory disturbances | 12  5.1% | 2  1.3% | 14  3.6% |
|  |  |  | Limitations in communication | 6  2.6% | 4  2.6% | 10  2.6% |
|  |  |  | Disturbances in mobility | 0 | 5  3.2% | 5  1.3% |
|  |  |  | Disease domains | 9  3.8% | 7  4.5% | 16  4.1% |
|  |  |  | Limitations in the basic and instrumental activities of life | 1  0.4% | 1  0.6% | 2  0.5% |
|  |  | Primary caregiver health | Physical well-being | 4  1.7% | 1  0.6% | 5  1.3% |
|  |  |  | Psychological well-being | 14  6% | 4  2.6% | 18  4.6% |
|  |  |  | Demands of care (for himself) | 6  2.6% | 2  1.3% | 8  2.1% |
|  |  |  | Factors affecting your health (related to the person with ND) | 4  1.7% | 4  2.6% | 8  2.1% |
|  |  |  | Factors affecting your health (not related to the person with ND) | 3  1.3% | 3  1.9% | 6  1.5% |
|  |  | Health of family members (other than primary caregiver) | Psychological well-being | 1  0.4% | 0 | 1  0.3% |
|  | **Total Family Health** | | | **44.4%** | **27%** | **31.5%** |
| Financial wellbeing | *But then, find a place and the economic issue. For example, I live on my pension.*  *We are going to identify it very well, with great effort. That is, the first, the economic aspect and the economic effort as it varies. I mean, from the beginning involved, I was involved with the center ten years ago, when I received grants of 60000 and 70000 euros via the Ministry and the Junta. How that has been transformed and, currently, I don't know for sure, but it is less than half.* | Financial resources for special care | | 1  0.4% | 4  2.6% | 5  1.3% |
|  |  | Lack of sufficient financial resources for care | | 1  0.4% | 1  0.6% | 2  0.5% |
|  |  | Accessibility and adaptability of the home | | 0 | 2  1.3% | 2  0.5% |
|  | **Total Financial wellbeing** | | | **0.8%** | **4.5%** | **2.3%** |
| Family relations | *And we are four siblings who take care of her and, among us, we organize ourselves so that she has the best quality of life and so do we.*  *Well, [in your case], she is your wife, but he is my brother and I have siblings. And one brother understands it, but another does not and says: Oh, so quit. But how am I going to leave him? How am I going to leave him if he's my brother?* | Family atmosphere | | 11  4.7% | 1  0.6% | 12  3.1% |
|  |  | Mutual support | | 7  3% | 8  5% | 15  3.9% |
|  |  | Family crisis | | 1  0.4% | 1  0.6% | 2  0.5% |
|  | **Total Family relations** | | | **8.1%** | **6.2%** | **7.5%** |
| Support from others | *And the daughters-in-law, who are two angels whom I received.*  *Just as I tell you that the family ehas brought us together a lot, I tell you that friends have fled.* | Emotional support from relatives, friends, and neighbors | | 0 | 1  0.6% | 1  0.3% |
|  |  | Practical support from relatives, friends, and neighbors | | 1  0.4% | 0 | 1  0.3% |
|  |  | Social isolation | | 2  0.8% | 1  0.6% | 3  0.8% |
|  | **Total Support from others** | | | **1.2%** | **1.2%** | **1.4%** |
| Support from services | *-The center, what [name of the P7] says is very good, but it isn't enough*  *-Of course, it isn't enough.*  *If, for example, in this rural area, because we are going to consider that we are in a rural area or the environment in the provincial setting, if instead of having a respite unit, we could have a day centre, it would undoubtedly improve the stay of the patient and the family member.*  *In my case, if something happens to me and of course [she] has to go to a residence, because my daughters, although they are all working in Madrid, they have to pay for a flat, they have to pay for school ... it's so impossible, so impossible* | Barriers to access to services | Barriers related to rural areas | 5  2% | 0 | 5  1.3% |
|  |  |  | Cost of services | 3  1.3% | 1  0.6% | 4  1% |
|  |  |  | Inadequate services | 3  1.3% | 1  0.6% | 4  1% |
|  |  |  | Insufficient services | 7  3% | 8  5% | 15  3.9% |
|  |  |  | Long waiting times to access services | 8  3.4% | 2  1.3% | 10  2.6% |
|  |  |  | Suggestions for improvement of services | 17  7.3% | 5  3.2% | 22  5.7% |
|  |  | Effects of services on FQoL | Improvement of FQoL | 2  0.9% | 1  0.6% | 3  0.8% |
|  |  |  | Social level | 1  0.4% | 1  0.6% | 2  0.5% |
|  |  | Health care for the person with ND and their family |  |  |  |  |
|  |  |  | Primary care team | 3  1.3% | 2  1.3% | 5  1.3% |
|  |  |  | Information about the diagnosis and progression of the ND | 0 | 1  0.6% | 1  0.3% |
|  |  |  | Information about the care needs of the person with ND | 1  0.4% | 1  0.6% | 2  0.5% |
|  |  |  | Specialized health care services | 10  4% | 8  5% | 18  4.6% |
|  |  | Social care for the person with ND and their family | Psychological support for the family | 3  1.3% | 0 | 3  0.8% |
|  |  |  | Technical support to the family | 1  0.4% | 0 | 1  0.3% |
|  |  |  | Associations | 8  3.4% | 8  5% | 8  2.1% |
|  |  |  | Care in centers for people in a situation of rest dependency of the caregiver | 11  4.7% | 1  0.6% | 12  3.1% |
|  |  |  | Comprehensive care in residential center | 5  2.1% | 2  1.3% | 7  1.8% |
|  |  |  | Home help | 1  0.4% | 0 | 1  0.3% |
|  |  |  | Hiring a caregiver | 1  0.4% | 0 | 1  0.3% |
|  |  |  | Public social services | 2  0.8% | 0 | 2  0.5% |
|  | **Total Support from Services** | | | **38.8%** | **26.3%** | **32.7%** |
| Influence of values | *I had a hard time taking this step, really, the first day, I had the impression that I wanted to get rid of it and the day I came back crying without him seeing me and then they say that to you as if they thought he was in the way.*  *We are not believers and we have not been envious of those who believe nor have we thought that whatever comes, comes, and that we must be fighters and that's all.* | Spiritual support that the family receives from the community | | 0 | 2  1.3% | 2  0.5% |
|  |  | Cultural beliefs that affect the whole family | | 1  0.4% | 0 | 1  0.3% |
|  |  | Personal beliefs that affect the whole family | | 4  1.7% | 2  1.3% | 6  1.5% |
|  |  | Religious beliefs and practices as a source of self-care | | 0 | 3  1.9% | 3  0.8% |
|  | **Total Influence of values** | | | **2.1%** | **4.5%** | **3.1%** |
| Careers and planning for careers | *It did affect me, not in the past, in the present because, for example, I have a store, how has it affected me? For example, when he started going to caregiver therapy sessions it was on Monday afternoons and I was going for quite some time, maybe two years...*  *You said he had been a teacher, a teacher of yours and he began to manifest a speech deficiency. He noticed that, in the classes, he did not have the fluency that he had had before, because up to that point, he had a very, very fluid capacity for expression, both in the classes and in the lectures.* | Effect of ND on the training or work activities of the person with ND | | 0 | 11  0.6% | 1  0.3% |
|  |  | Effect of EN on the work activity of a family member | | 0 | 2  1.3% | 2  0.5% |
|  | **Total Careers and planning for careers** | | | **0%** | **1.9%** | **0.8%** |
| Leisure and recreation | *I really like dancing and concerts and said 'We can't leave grandma alone , well I took her along and she ended up liking it, she came with another person.... and also [with] me.*  *We just stopped going to the movies, yes. Because he said: No more.* | Abandonment of leisure and free time activities | | 1  0.4% | 3  1.9% | 4  1% |
|  |  | Primary caregiver rest | | 3  1.3% | 0 | 3  0.8% |
|  |  | Leisure and free time with the family | | 0 | 6  3.9% | 6  1.5% |
|  |  | Leisure and free time of family members (beyond family) | | 0 | 7  4.5% | 7  1.8% |
|  | **Total Leisure and recreation** | | | **1.7%** | **10.3%** | **5.1%** |
| Community interaction | *For example, I often avoid going for a walk where there are people. It's because I see that they're harming my wife.*  *We don't know how to deal with it and, at the end of the day, I don't know why we've isolated them. It's as if we pushed Alzheimers away. If these people are integrated, these people, why do we isolate them? That is what I do not understand. Why are there no campaigns about Alzheimer's, but here too there is no training in any of this.* | Family relationships with their close environment | | 7  3% | 3  1.9% | 10  2.6% |
|  |  | Acceptance of the family by the people around them | | 12  5.1% | 5  3.2% | 17  4.4% |
|  | **Total Community interaction** | | | **8.1%** | **5.1%** | **7%** |
| Others | *When I talk about dependent people, they are indeed dependents and, as dependents, it is 24 hours or 18 hours that we are awake, but it is an interdependent dependency. They depend on us, we are not dependent on them. But already the relationship is obviously affective. It's an uncontaminated connection. I mean, the connection that we have with the care of our family members is excessive.* | Related to gender | | 1  0.4% | 1  0.6% | 2  0.5% |
|  |  | Love and affection as an explanation of the care | | 2  0.9% | 1  0.6% | 3  0.8% |
|  | **Total Others** | | | **1.3%** | **1.2%** | **1.3%** |
| **TOTAL** | | | | **234** | **155** | **389** |
